# Bacterial Lipopolysaccharide Augments Febrile-Range Hyperthermia-Induced Heat Shock Protein 70 Expression and Extracellular Release in Human THP1 Cells

**DOI:** 10.1371/journal.pone.0118010

**Published:** 2015-02-06

**Authors:** Mohan E. Tulapurkar, Aparna Ramarathnam, Jeffrey D. Hasday, Ishwar S. Singh

**Affiliations:** 1 Division of Pulmonary and Critical Care, Department of Medicine, University of Maryland School of Medicine, Baltimore, Maryland, United States of America; 2 Cytokine Core Laboratory, University of Maryland School of Medicine, Baltimore, Maryland, United States of America; 3 Research Services of the Baltimore Veteran Affairs Medical Center, Baltimore, Maryland, United States of America

## Abstract

Sepsis, a devastating and often lethal complication of severe infection, is characterized by fever and dysregulated inflammation. While infections activate the inflammatory response in part through Toll-like receptors (TLRs), fever can partially activate the heat shock response with generation of heat shock proteins (HSPs). Since extracellular HSPs, especially HSP70 (eHSP70), are proinflammatory TLR agonists, we investigated how exposure to the TLR4 agonist, bacterial lipopolysaccharide (LPS) and febrile range hyperthermia (FRH; 39.5°C) modify HSP70 expression and extracellular release. Using differentiated THP1 cells, we found that concurrent exposure to FRH and LPS as well as TLR2 and TLR3 agonists synergized to activate expression of inducible HSP72 (HSPA1A) mRNA and protein via a p38 MAP kinase-requiring mechanism. Treatment with LPS for 6 h stimulated eHSP70 release; levels of eHSP70 released at 39.5°C were higher than at 37°C roughly paralleling the increase in intracellular HSP72 in the 39.5°C cells. By contrast, 6 h exposure to FRH in the absence of LPS failed to promote eHSP70 release. Release of eHSP70 by LPS-treated THP1 cells was inhibited by glibenclamide, but not brefeldin, indicating that eHSP70 secretion occurred via a non-classical protein secretory mechanism. Analysis of eHSP70 levels in exosomes and exosome-depleted culture supernatants from LPS-treated THP1 cells using ELISA demonstrated similar eHSP70 levels in unfractionated and exosome-depleted culture supernatants, indicating that LPS-stimulated eHSP70 release did not occur via the exosome pathway. Immunoblot analysis of the exosome fraction of culture supernatants from these cells showed constitutive HSC70 (HSPA8) to be the predominant HSP70 family member present in exosomes. In summary, we have shown that LPS stimulates macrophages to secrete inducible HSP72 via a non-classical non-exosomal pathway while synergizing with FRH exposure to increase both intracellular and secreted levels of inducible HSP72. The impact of increased macrophage intracellular HSP70 levels and augmented secretion of proinflammatory eHSP70 in the febrile, infected patient remains to be elucidated.

## Introduction

Sepsis is a devastating, often lethal complication of severe infection and injury, characterized by excessive and dysregulated inflammation, multi-organ injury and cardiovascular collapse. The incidence of severe sepsis is between 300 and 1031 cases per 100,000, depending on the definitions and methods used and, despite myriad basic and clinical research studies, in-hospital mortality remains between 14.7 and 29.9% [1]. Most patients with sepsis are febrile and, Schortgen *et al*. [2] recently showed that aggressive fever management reduced the duration of shock and 14-day mortality in patients with septic shock. Although the mechanisms by which fever reduction improves mortality in septic shock are not yet well understood, fever and febrile-range hyperthermia (FRH) is known to exert many biological effects that may impact on survival during sepsis, including partial activation of the heat shock response (HSR).

Activation of the HSR stimulates generation of heat shock proteins (HSPs), which participate in the regulation of various pathophysiological functions [3, 4]. Mammals express many HSPs that are grouped within five conserved families according to their molecular weight. Among mammalian HSPs, inducible HSP70 (also called HSP72), which is coded by two almost identical genes, *hspa1a* and *hspa1b*), is the most widely studied member. HSP72 is strongly induced following exposure to stress by the heat/stress-activated transcription factor, heat shock factor-1 (HSF1). HSF-1 undergoes stepwise activation in response to stress that includes trimerization in the cytoplasm, nuclear translocation and multiple serine/threonine phosphorylation events to attain HSP gene activating activity [5–7]. We have previously shown that HSF1 activation and induction of HSP gene expression occurs at temperatures within the usual febrile range (38.5–40°C) [8]. We and others have shown that HSF1 and certain HSPs can modify innate immune responses, which may shape the inflammatory response during febrile illnesses such as sepsis, trauma, cancer, and drug transfusion reactions (reviewed in [9, 10]), or during hyperthermic states including exertional/environmental or malignant hyperthermia [9–12].

The role of HSPs, especially HSP70, in sepsis and associated organ injury is complex because of their pleiotropic effects on cell survival and inflammation, depending on whether they accumulate intra- or extra-cellularly. Whereas intracellular HSP70 acts as chaperones that exert anti-apoptotic and cytoprotective actions [7, 13], extracellular HSP70 (eHSP70) is immunomodulatory. Acting as a TLR agonist, eHSP70 not only promotes inflammation [14], but its persistent presence may eventually cause immune suppression by inducing tolerance to TLR agonists [15], two hallmarks of severe sepsis. Unfortunately, few studies have addressed the mechanism of eHSP70 release in infection and sepsis. Available data suggest that proinflammatory agonists contribute to increased HSP70 gene expression and extracellular release, which may explain the elevated circulating levels of eHSP70 found in patients with ARDS and severe sepsis [16, 17]. Treatment with LPS or proinflammatory mediators like IL-6 and TNFα induce HSP expression in human lung pericytes, HuH7 hepatoma cells, peripheral blood mononuclear cells and in adult feline cardiac myocytes [18–20]. In our earlier study we showed that TLR agonists and FRH (39.5°C) synergized to increase HSP70 gene expression and eHSP70 release in the RAW264.7 mouse macrophage cell line [21]. We showed that the effect was mediated at least in part by p38 MAP kinase dependent phosphorylation of histone H3 and increased recruitment of HSF1 to the HSP70 chromatin [21]. In the present paper we have extended our prior studies by demonstrating similar synergy between LPS and FRH for HSP70 expression and release in the THP1 human monocyte/macrophage cell line, showing that this effect was unique to HSP70 and not shared by HSP90 or HSP110, and that the extracellular release of HSP70 stimulated by LPS FRH was mediated via a non-classical, non-exosomal protein secretion pathway.

## Materials and Methods

### Cell culture

The THP1 human monocyte cell line (ATCC TIB202) was maintained in RPMI 1640 supplemented with 2 mM L-glutamine, 1 mM sodium pyruvate, 10 mM HEPES buffer, pH 7.3, 0.05mM β-mercaptoethanol and 10% defined fetal bovine serum (all from Gibco, Life Technologies, Grand Island, NY). Cells were routinely tested for Mycoplasma infection using a commercial assay system (MycoTest, Invitrogen), and new cultures were established monthly from frozen stocks. Cell viability was determined by trypan blue dye exclusion.

Prior to experimental exposures, THP1 cells were differentiated by treating with 50 ng/ml Phorbol 12-myristate 13-acetate (PMA, Sigma-Aldrich) for 24 h, washing with PBS, and culturing at 37°C in PMA-free media for an additional 24 h. The complete RPMI growth media was then removed, the cells washed twice in PBS and Opti-MEM reduced serum medium (Gibco, Life Technologies) was added to reduce background HSP70 and exosome levels that are contributed by serum. For FRH exposure, cells were incubated at 39.5°C for the indicated time in automatic CO_2_ incubators certified to have <0.2°C temperature variation (Forma; Marietta, OH) and calibrated for each experiment using an electronic thermometer (FLUKE Instruments model 5211, Everett, WA). For LPS stimulation, cells were treated with LPS from E. coli 0111B4 (Sigma Aldrich; St. Louis, MO) and maintained at either 37°C (normothermic control) or at 39.5°C (FRH) as indicated. TLR2 and-3 agonists, Pam3cys and poly IC, respectively were obtained from Invivogen (San Diego, CA) and used as indicated for LPS.

### RNA Extraction and quantitative real-time PCR

Total RNA from differentiated THP1 cells was isolated using Qiagen spin-columns, contaminating DNA was eliminated by digesting with DNase I (Promega) and RNA was reverse-transcribed using oligo-dT primers and a cDNA synthesis kit according to the manufacturer’s protocol (Promega) as we have previously described [21–23]. Duplicate 25 μl real-time PCR reactions were performed in 96 well plates using a SYBR-Green reaction mix (BioRad) and a BioRad iCycler IQ Optical Module according to the supplier’s protocol with the following forward and reverse primers: glyceraldehydes-3-phosphate dehydrogenase (GAPDH), 5’-agcctcgtcccgtagacaaaat and 5’-tggcaacaatctccactttgc; HSP72 (HSPA1A, NM_005345), 5’-ggccagggctggattact and 5’-gcaaccaccatgcaagatta; HSP90 (HSPAA1, NM_005348), 5′-tgcggtcacttagccaagatg and 5′-gaaaggcgaacgtctcaacct; and HSP110 (HSPH1, NM_006644), 5′-gctacacgaattccagctgtga and 5′gagcagcatggtttcgactaaa. Data were quantified using the Gene Expression Ct Difference method and standardized to levels of the housekeeping gene, GAPDH, using Ct values automatically determined by the thermocycler [21–23].

### Immunoblotting

Cell extracts were prepared in lysis buffer containing 10 mM Tris-HCl, pH 7.4, 150 mN NaCl, 0.1% TritonX-100 and protease and phosphatase inhibitors (Boehringer Mannheim) and protein content was assayed by the Bradford method (BioRad) using a bovine serum albumin standard curve as described earlier [21, 22]. Lysates containing 10–20μg total protein per lane were resolved by SDS-PAGE, electrostatically transferred to polyvinylidene difluoride membrane and blocked with Odyssey Blocking Buffer (Li-COR Biosciences, Nebraska USA). The blots were probed with the indicated primary antibodies in TTBS (50 mM Tris, 150 mM NaCl, 0.05% Tween 20) followed by incubation with the secondary antibody, IRDye 800 goat anti-mouse or IRDye 680 Goat anti-Rabbit IgG (1:15000, Li-COR Biosciences) as applicable and resultant bands was visualized using the Odyssey Infrared Imaging System, v3.0 (Li-COR Biosciences, Nebraska USA). Primary antibodies used were as follows: inducible HSP70 (HSPA1A) (1:10000; SPA-812, Enzo), total HSP70 (1:5000; H5147, Sigma), CD63 (1:1000; SantaCruz), β-tubulin (1:10000; Chemicon), and total and phospho-p38 (both 1:1000; Cell Signaling).

### Exosome isolation

Exososmes were isolated by differential centrifugation [24]. Briefly, cell culture media from treated THP1 cells were collected and centrifuged at 4°C at 3000g for 10 min to remove floating cells and cellular debris. The supernatants were then centrifuged at 20,000xg for 30 min at 4°C. The post 20,000g supernatant was then spun at 100,000xg for 1 h, the exosomal pellet was washed by suspending in 1 ml PBS and re-centrifuged at 100,000xg for 1 h and the resulting exosomal pellet was resuspended in 50ul of PBS and stored at -80°C for further analysis. For exosome depleted cell culture media, the post 100,000g supernatant was collected and analyzed as indicated.

### HSP70 ELISA

THP1 cells were treated with or without LPS and incubated at 37°C or 39.5°C for 6 h or 24 h as indicated. Cell culture supernatants were collected, centrifuged for 10 min at 3000g to remove floating cells and debris, and stored at -80°C. Supernatants were analyzed for HSP70 by ELISA using a Human/Mouse/Rat Total HSP70 DuoSet IC ELISA Kit (DYC1663E, R & D Systems; Minneapolis, MN) at the University of Maryland Cytokine Core Laboratory as previously described [21]. The assay recognizes both inducible and constitutive HSP70 isoforms and has a lower detection limit of 156 pg/ml.

### Statistical analysis

Data are displayed as mean ± SE. Differences between two groups were analyzed by unpaired Student t-test and among multiple groups was analyzed by applying a post-hoc Tukey-Kramer Honestly Significant Difference test to a one-way ANOVA. Differences with p < 0.05 were considered to be statistically significant.

## Results

### TLR agonists enhance FRH-induced HSP70 (HSPA1A) expression in THP1 cells

**Fig 1:**
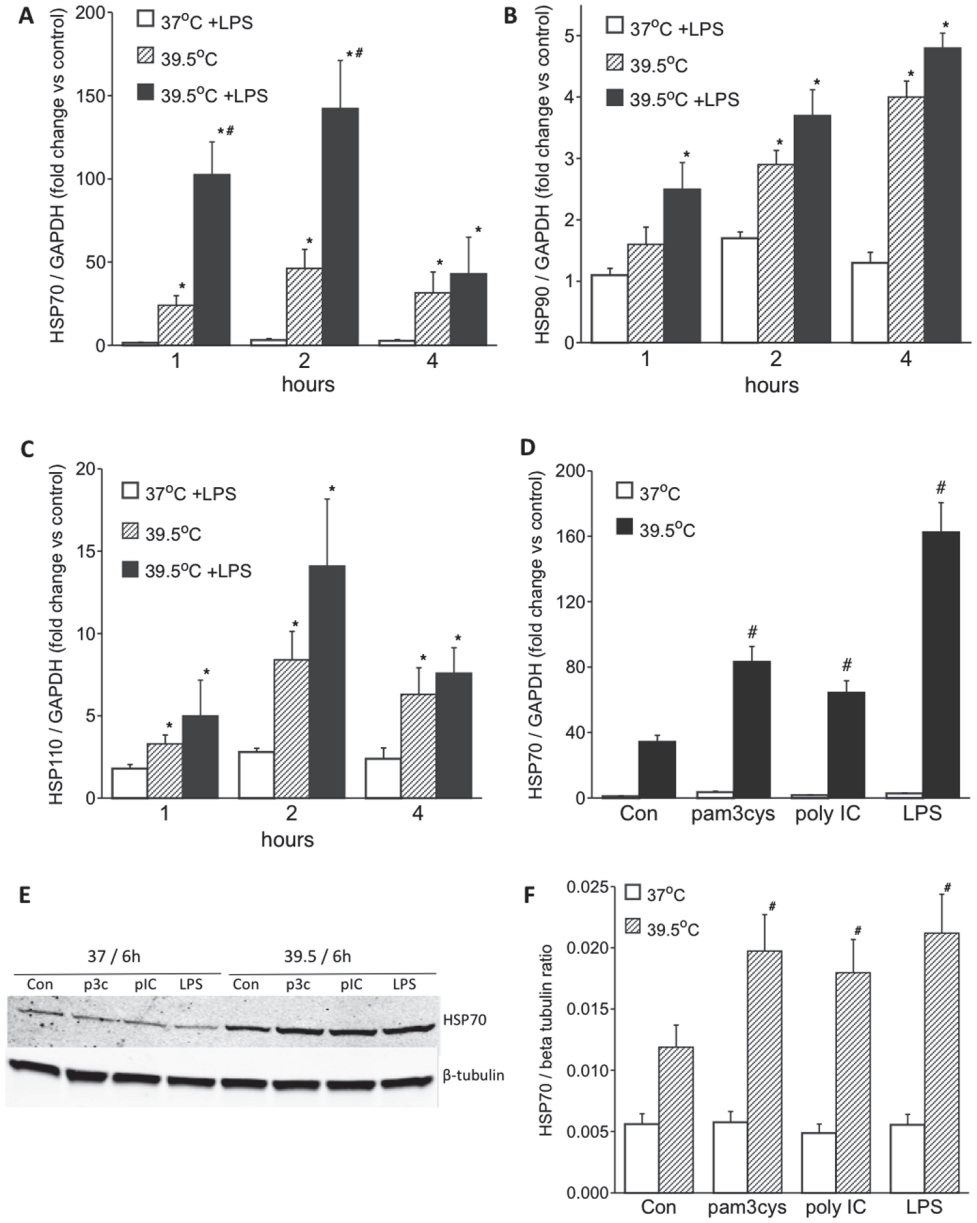
TLR agonists enhance FRH-induced HSP70 expression in THP1 *cells*: A–C. Differentiated THP1 cells were stimulated with 100 ng/ml LPS at 37°C or 39.5°C for 1, 2 or 4 h, and HSP70 (HSPA1A) (A), HSP90 (HSP90AA1) (B), and HSP110 (HSPH1) (C) mRNA levels were measured by qPCR, normalized to GAPDH, and expressed as fold change vs. levels in 37°C untreated controls. D. Differentiated THP1 cells were incubated with 0.5 μg/ml Pam3CSK4, 12.5 μg/ml poly IC, or 100 ng/ml LPS for 2 h and HSP70 mRNA levels measured. E. Differentiated THP1 cells were incubated with 0.5 μg/ml Pam3cys (p3c), 12.5 μg/ml poly IC (p IC), or 100 ng/ml LPS for 6 h at 37°C or 39.5°C and cell lysates were immunoblotted for inducible HSP72 using SPA-812 anti-HSP70 antibody F. The HSP72 band intensites were normalized to ß-tubulin and expressed as fold-change compared with untreated 37°C controls. Data are mean±SE from 4 independent experiments. * and # denote p<0.05 vs. 37°C controls and LPS-untreated 39.5°C cells, respectively.

To analyze the effects of LPS on FRH-induced HSP expression, we measured HSP72 (HSPA1A, NM_005345). HSP90 (HSP90AA1, NM_005348) and HSP110 (HSPH1, NM_006644) mRNA levels in THP1 cells co-exposed to LPS and FRH. PMA-differentiated THP1 cells were stimulated with 100 ng/ml LPS and incubated either at 37°C or at 39.5°C for 1, 2 or 4 h. In the absence of TLR agonists, incubating THP1 cells at 39.5°C stimulated a 20–40 fold increase in HSP70 (HSPA1A) mRNA levels compared with cells incubated at 37°C. Stimulating cells with LPS at 37°C failed to induce HSPA1A expression, but co-exposure to LPS and 39.5°C increased HSPA1A mRNA levels to 100–140-fold greater than untreated 37°C cells and 4–5 fold greater than cell incubated at 39.5°C without TLR agonist (Fig. 1a). Exposing THP1 cells to 39.5°C was also sufficient to induce expression of both HSP90 (Fig. 1b) and HSP110 (Fig. 1c) mRNA, but in contrast with HSPA1A, co-exposure to LPS had no effect on HSP90 or HSP110 expression in 39.5°C THP1 cultures. To determine whether activation of other TLRs would also synergize with FRH to induce HSPA1A expression, we analyzed HSPA1A mRNA expression level in THP1 cells incubated with a TLR1/2 agonist (Pam3cys; 0.5 μg/ml) or TLR3 agonist (poly IC; 12.5 μg/ml) at 37°C or at 39.5°C for 2 h (Fig. 1d). Both Pam3Cys and poly IC exerted effects similar to LPS, greatly augmenting HSPA1A mRNA levels in 39.5°C THP1 cell culture. To confirm that the synergistic induction of HSPA1A mRNA levels by TLR activation and FRH exposure was translated to the protein level, we analyzed inducible HSP72 protein levels in THP1 lysates by immunoblotting. THP1 cells were treated with LPS, poly IC, or Pam 3cys at 37°C or at 39.5°C as was done for the mRNA analysis except the cells were incubated for 6h prior to lysis and immunoblotting for inducible HSP70 (anti-HSPA1A antibody SPA-812, Enzo Life Sciences) (Fig. 1e, f). As expected FRH exposure caused a 2.5-fold increase in HSPA1A expression and treatment with each of the three TLR agonists stimulated a 1.5–2-fold additional increase in HSPA1A protein expression, but failed to induce an increase in HSPA1A protein levels in 37°C THP1 cultures.

### p38 MAPK inhibitor blocks LPS-induced increase in HSP70 production in FRH-exposed THP1 cells

**Fig 2:**
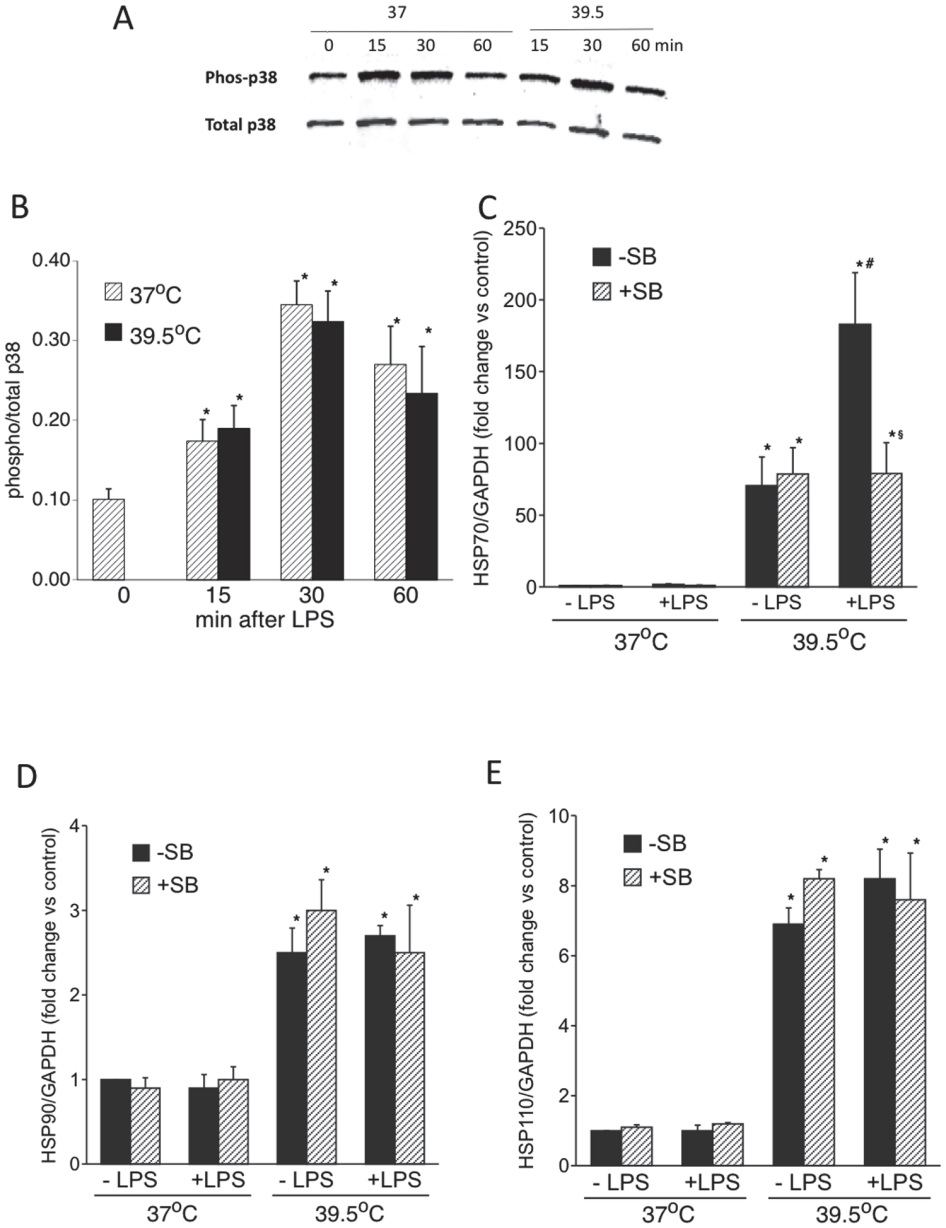
p38 MAPK inhibitor inhibits LPS-induced increase in HSP70 levels in FRH-exposed THP1 cells: A, B. Differentiated THP1 cells were incubated with or without 100 ng/ml LPS at 37° or 39.5°C. Cells were lysed at the indicated time and immunoblotted for phosphorylated and total p38α/β. A representative blot and mean ± SE of band densities from four experiments plotted as the ratio of phosphorylated to total p38 are shown in A and B, respectively. C–E. Differentiated THP1 cells were pretreated with either 0.1% DMSO or 10 μM SB203580 HCl (SB) in DMSO for 30 min and then stimulated with 100 ng/ml LPS for 2 h. RNA was isolated and HSP70 (HSPA1A) (B), HSP90 (HSP90AA1) (C), and HSP110 (HSPH1) (D) mRNA was quantified by qPCR, normalized to GAPDH, and expressed as fold change vs. untreated 37°C control. Data presented as mean+SE, n = 4. *, # and § denote p<0.05 vs. 37°C controls, LPS-untreated 39.5C cells, and SB203580-untreated cells, respectively.

In an earlier study [21] we showed that p38-MAPK inhibition blocked the augmentation of HSPA1A expression by LPS in FRH-exposed RAW cells. To confirm that p38 MAPK signaling also participated in TLR-dependent HSPA1A gene regulation in human mononuclear phagocytes, we first analyzed p38-MAPK activation by immunoblotting for the dual phosphorylated active form. As was seen in RAW 264.7 cells, treatment with LPS stimulated similar activation of p38-MAPK in both 37°C and 39.5°C THP1 cell cultures (Fig. 2a, b). To determine whether p38-MAPK activity was required for the augmented HSPA1A expression stimulated by adding LPS-to 39.5°C THP1 cell cultures, we analyzed the effect of 30 min pretreatment with the p38 MAPK-α/β inhibitor SB203580 on HSPA1A mRNA expression. As we previously found in RAW cells, SB203580 but not DMSO (vehicle) abrogated the stimulatory effect of LPS on HSPA1A expression, but had no effect on induction of HSPA1A (Fig. 2c), HSP90 (Fig. 2d), or HSP110 (Fig. 2e) in THP1 cells exposed at 39.5°C in the absence of LPS.

### LPS/FRH co-exposure increases secretion of eHSP70 via a non-classical protein secretory pathway

**Fig 3:**
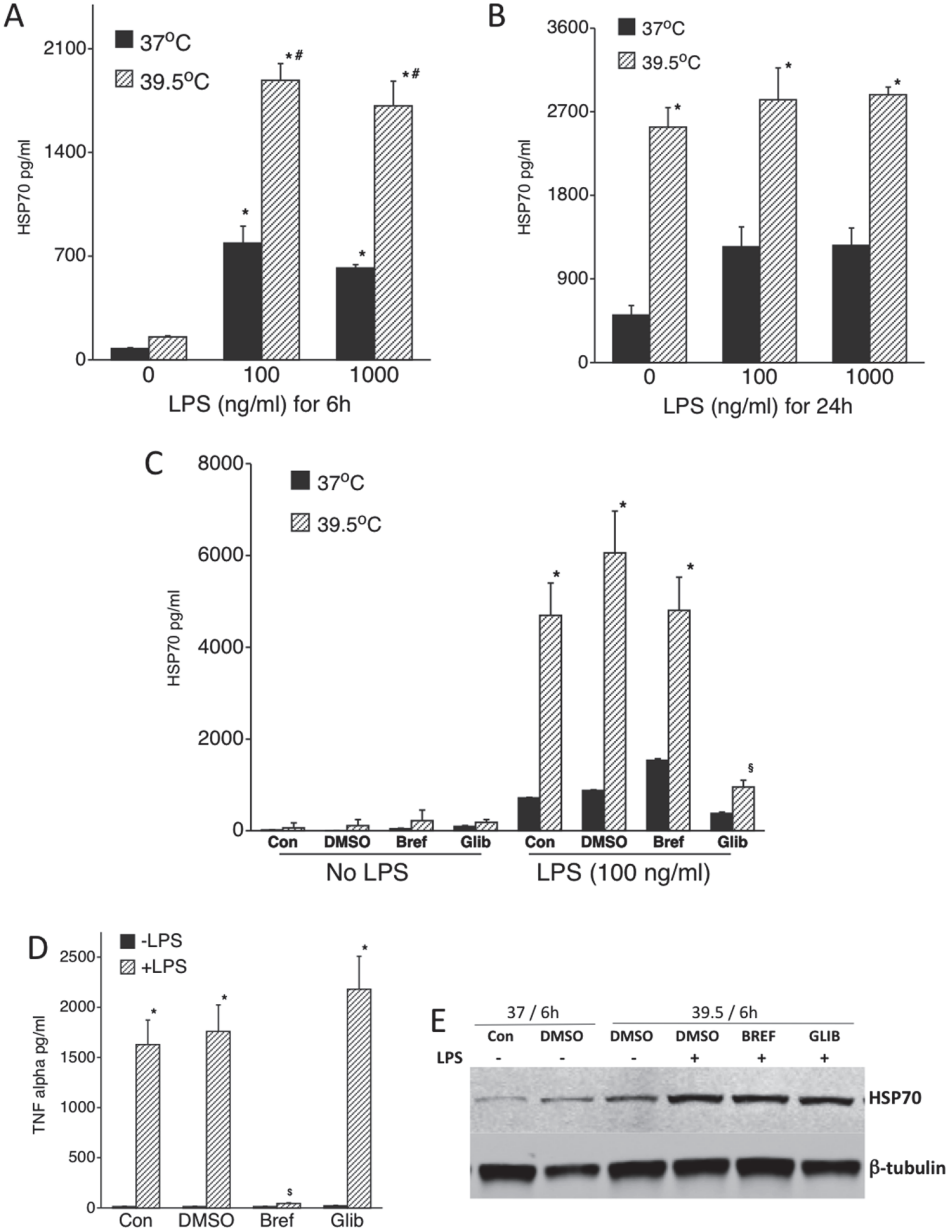
LPS/FRH co-exposure increases release of eHSP70 that is secreted via a non classical protein secretory pathway: A, B. Differentiated THP1 cells were stimulated with 100 ng/ml or 1 μg/ml of LPS as indicated and incubated either at 37°C or 39.5°C for 6 h (A) or 24h (B). Cell culture supernatants were collected and HSP70 levels measured by ELISA. (C) To analyze the effect of protein secretory pathway inhibitors, cells were treated with 0.1% (v/v) DMSO (vehicle), 5 μg/ml Brefeldin or 100uM Glibenclamide at 37°C for 1 h, then incubated with or without 100 ng/ml LPS for 6 h at 37°C or 39.5°C. The cell culture supernatants were collected and analyzed for HSP70 by ELISA. (D). The supernatants from the 37°C (with and without LPS) culture supernatants from panel C were analyzed for TNFα by ELISA. (E) A representative of four similar immunoblots of lysates from cells in panel C immunoblotted for inducible HSP72 and β-tubulin. Data presented as mean+SE, n = 4. *, and # denote p<0.05 vs. LPS-untreated and DMSO+LPS-treated cells, respectively.

We previously demonstrated in RAW 264.7 cells that co-exposure to LPS and FRH not only induces increased intracellular levels of HSP70 protein, but also stimulated release of HSP70 into the extracellular microenvironment [21] where it can exert its TLR4 agonist activity [14, 25]. To confirm that human mononuclear phagocytes increase release of HSP70 when co-stimulated with LPS and FRH, we stimulated THP1 cells with 100 ng/ml or 1 μg/ml LPS at 37°C or at 39.5°C for 6 or 24 h, removed cells by centrifugation at 3000g for 10 min, and measured HSP70 concentration in the cleared supernatant by ELISA. In the absence of LPS, incubation at 39.5°C for 6 h failed to stimulate HSP70 release, but LPS treatment for 6 h at either 37°C or 39.5°C increased eHSP70 release (Fig. 3a). In contrast, incubating THP1 cells at 39.5°C for 24 h caused similarly high release of eHSP70 whether or not LPS was present. Since HSP70 release appeared to occur through LPS-inducible secretion during 6 h incubation, while HSP70 more likely leaked from injured or dead cells after 24 h FRH exposure, we further studied the mechanisms of HSP70 secretion during 6 h FRH exposures.

To determine whether LPS stimulated eHSP70 secretion occurs via a classical or non-classical pathway of protein secretion, we pretreated THP1 cells with 5ug/ml brefeldin A or 100uM glibenclamide in 1 μl/ml DMSO, or 0.1% DMSO for 1 h at 37°C prior to stimulating with 100 ng/ml LPS at 37°C or at 39.5°C. Cell culture media were collected after 6 h and eHSP70 was measured by ELISA. Whereas pretreatment with brefeldin A failed to modify eHSP70 release compared with DMSO, pretreatment with glibenclamide significantly reduced HSP70 release (Fig. 3c). To confirm the activity of brefeldin A in these cells, we showed that it completely blocked LPS-induced TNFα secretion (Fig. 3d), which is known to be a brefeldin A-sensitive process [26]. In contrast with effects on secretion, neither brefeldin nor glibenclamide altered intracellular levels of HSP70 in these cells (Fig. 3e).

### The eHSP70 released from LPS/FRH-stimulated cells is predominantly inducible HSP70 (HSPA1A) rather than constitutive HSC70 (HSPA8) and is not associated with exosomes

**Fig 4:**
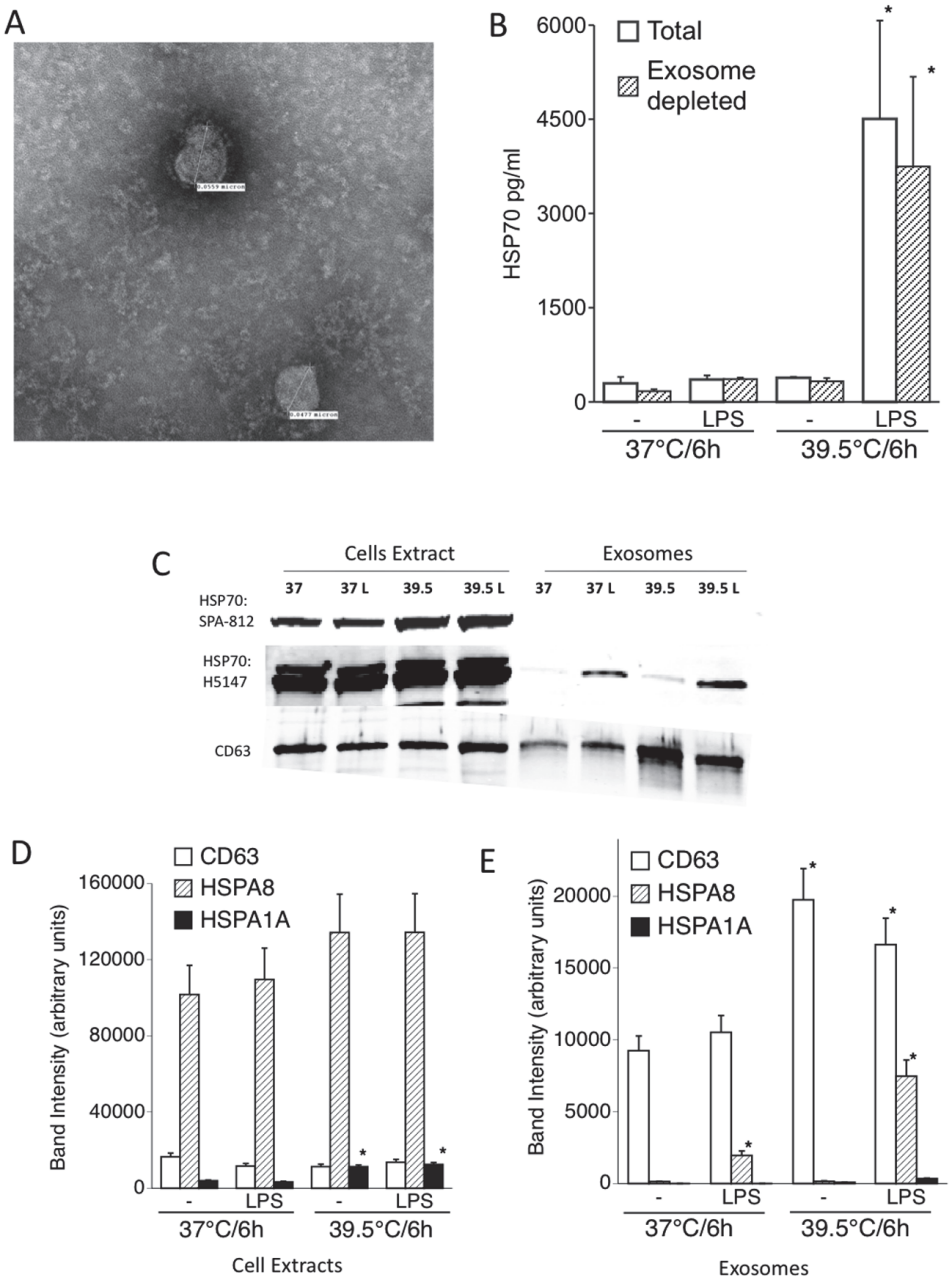
LPS/FRH-induced eHSP70 is predominantly the inducible form and is secreted independent of exosomes: Differentiated THP1 cells were incubated with or without 100 ng/ml LPS at 37°C or 39.5°C for 6 h, cell culture supernatants were collected and half of each were cleared of exosomes (esxosome-depleted) via ultracentrifugation; the remaining sample volume remained unfractionated (total). (A) electron micrograph of exosome fraction (B). HSP70 levels (including both inducible HSP72 and constitutive HSC70 forms) in the total and exosome-depleted cell culture supernatants was assayed by ELISA. (C–E) Total cell extracts and isolated exosomes from cells from panels A and B were immunoblotted using SPA-812 anti-HSP70 antibody (Enzo), which detects only inducible HSP72, H5147 anti-HSP70 antibody (Sigma-Aldrich), which detects both HSP72 and constitutive HSC70, and anti-CD63 antibody (C). Band intensities for CD63, HSPA8 and HSPA1A were quantified and plotted for cell extracts (D) and exosomes (E). Data presented as mean+SE, n = 6. * denotes p<0.05 vs. controls. Images presented are representative of 3 or more independent experiments.

Previous studies in human peripheral blood mononuclear cells (PBMCs) and rat endothelial cells have shown that eHSP70 is secreted via exosomes [27, 28] and HSP70 is usually found at high levels in exosomal preparations [29, 30]. To determine whether the LPS-induced eHSP70 release in FRH-exposed cells is mediated via the exosomal route, we analyzed whether depleting THP1-conditioned medium of exosomes would reduce their eHSP70 content. Cell culture media from THP1 cells incubated for 6 h with or without LPS at 37° or 39.5°C were sequentially centrifuged, at 3000g for 10 min to remove cells and debris, at 13,000g for 30 min to isolate microvesicles, and at 100,000g for 1h to sediment exosomes and the final supernatants were collected as exosome-depleted media. Sedimented exosomes were verified by their characteristic size and appearance by electron microscopy (Fig. 4a). HSP70 levels in the exosome-sufficient post-3000g and exosome-depleted post-100,000g supernatants were measured by ELISA (Fig. 4b). For each of the four treatments, the exosome-sufficient and exosome-depleted culture media had similar HSP70 content. To analyze the HSP70 content of the exosomes, we immunoblotted the isolated exosomes and the corresponding cell lysates for HSP70 using two different anti-HSP70 antibodies. Using antibody SPA-812 (Enzo) which exclusively identifies inducible HSP72 (coded for by *hspa1a* and *hspa1b*) demonstrated a marked increase in HSP72 band intensity in cell extracts from 39.5°C- and LPS/39.5°C-exposed THP1 cells (Fig. 4c, d), but failed to detect HSP72 in the isolated exosomes (Fig. 4c, e). In contrast, antibody H5147 (Sigma), which recognizes both the constitutive (HSC70, HSPA8) and inducible forms of HSP70, showed distinct bands in the isolated exosomes, especially in exosomes isolated from LPS-treated 37° and 39.5°C THP1 cells (Fig. 4c, e). Immunblotting with an antibody against the exosome marker CD63 confirmed the presence of exosomes and demonstrated a modest increase in exosome release during 39.5°C incubation in the absence or presence of LPS.

## Discussion

HSPs were initially described as evolutionarily conserved cytoprotective proteins and the HSR as a binary biological response induced by exposure to stress, including high temperatures and orchestrated in eukaryotic cells by HSF1 [3]. More recently, studies from our group and others have shown that HSF1 can be activated by less extreme hyperthermia, including temperatures within the normal febrile range [8, 31], that some inflammatory mediators such as type I interferons and arachidonic acid can lower the thermal threshold for HSF1 activation [32], and that HSF1 and other elements of the HSR can exert potent immunomodulatory effects [9, 10, 22, 23, 31, 33–42]. Inducible HSP72 (HSPA1A) is the predominant HSP generated by mammalian cells in response to heat stress [43]. HSP70 can be released from cells and has been found in the circulation of patients with infection, sepsis and trauma, and in aging subjects [44–48]. Since intracellular HSP70 is cytoprotective [7, 13] and extracellular HSP70 is proinflammatory [14] and both can occur during febrile illnesses it is important to understand both mechanisms of HSP gene activation and extracellular release at febrile temperatures.

We previously showed in the mouse RAW264.7 macrophage cell line *in vitro* and in a mouse intratracheal LPS-induced lung injury model *in vivo* that multiple TLR agonists and interleukin-1ß synergizes with FRH to enhance HSP72 expression and extracellular release of HSP70 without loss of plasma membrane integrity, suggesting active secretion. We further demonstrated that activation of HSP72 expression in RAW264.7 cells was p38 MAPK-dependent and associated with p38-dependent histone H3 phosphorylation and enhanced recruitment of HSF1 to the HSPA1A chromatin [21]. In the present paper we have extended these findings by showing that FRH and TLR agonists also synergize to increase HSP72 expression and extracellular release in the THP1 human macrophage cell line through a p38-dependent process and that LPS activates HSP70 release through a non-classical, glibenclamide-sensitive secretion mechanism.

As was found in RAW264.7 cells, treatment of THP1 cells with LPS was not sufficient to activate HSPA1A gene expression at 37°C, but exposing the cells to FRH (39.5°C) alone caused a 20–40 fold increase in HSPA1A mRNA and LPS stimulated a further 4–5-fold increase (Fig. 1). Moreover, like RAW 264.7 cells, pretreating THP1 cells with SB203580, a pharmacologic inhibitor of p38α/β, blocked the effects of LPS but not FRH on HSPA1A expression (Fig. 2). These data suggest that LPS and FRH exert effects on HSPA1A gene expression through distinct signaling pathways that converge on the interaction of activated HSF1 and the HSPA1A promoter. LPS modifies HSPA1A expression through p38 MAPK signaling but only in the presence of FRH. In a previous study we showed that the augmentation of HSPA1A expression by LPS was associated with p38-dependent phosphorylation of promoter-associated histone H3 and recruitment of HSF1 to the HSPA1A promoter [21]. Whether this is the sole mechanism by which p38 modifies HSPA1A expression is not yet known.

We have previously shown that exposing human A549 lung epithelial cells to 38.5°, 39.5°, and 41°C induces similar 3-fold increase in levels of the trimeric DNA-binding form of HSF1 but only modest HSPA1A gene expression [8]. Increasing temperature further from 41° to 42°C increased HSPA1A gene expression by 14-fold despite only increasing levels of trimerized HSF1 by only an additional 50%. However, increasing incubation temperature from 41° to 42°C did stimulate a marked decrease in the electrophorectic mobility of HSF1 in SDS-PAGE, suggesting extensive post-translational modification [8]. We have previously shown in RAW264.7 cells that stimulation with LPS at 37°C was sufficient to cause HSF1 post-translational modifications and decreased HSF1 electrophoretic mobility but without activating HSP70 gene expression [21]. These data suggest that LPS can augment HSPA1A expression via p38 MAPK activation and chromatin modifications that increase access of activated HSF1 to the HSPA1A promoter, but only in the setting of FRH. These data suggest that LPS may cause additional modifications to trimerized, but not monomeric HSF1 that increases its transcriptional activating activity. We have previously shown that FRH exposure is sufficient to cause a relatively slow p38 activation in the absence of a second signal [38]; however, the failure of SB203580 to block HSPA1A, HSPAA1 or HSPH1 gene expression in LPS-free 39.5°C THP1 cell culture suggests that FRH-induced HSP gene activation is independent of p38 MAPK activation.

Studies by several groups showed that HSP70 is released from cells via both necrosis and by active secretion [49, 50]. While a non-classical secretion pathway is generally agreed upon, several different mechanisms have been proposed, including release by secretory-like granules [51], via an ATP binding cassette (ABC) transport-like system [50, 52], or by its insertion into membrane of export vesicles [49, 53]. We previously showed that co-exposing RAW 264.7 cells to LPS and FRH, but not either stimulus alone caused extracellular release of HSP70 but not LDH [21], suggesting HSP70 secretion rather than release from injured cells. In this study, LPS stimulated marked increases in release of HSP70 in both 37°C and 39.5°C THP1 cell cultures, whereas THP1 cells incubated at 39.5°C without LPS released only low levels of HSP70, suggesting that HSP70 release is an LPS-dependent process. To further define the mechanism of HSP70 secretion, we used a pharmacologic inhibitor of classical protein secretion, brefeldin A and an inhibitor of ABC family transporter activity, glibenclamide. Pretreatment with brefeldin A failed to alter or modify LPS-induced HSP70 release, but did abrogate secretion of TNFα, which is known to utilize the classical secretory pathway [26]. In contrast, pretreatment with glibenclamide potently inhibited LPS-induced HSP70 release at both 37° and 39.5°C. These results support a non-classical secretory pathway for HSP70 and are consistent with its lack of a consensus signal sequence and with previous studies from the Calderwood group that showed an endolysosomal mechanism involving the ATP binding cassette (ABC) family transporter proteins in the secretion of HSP70 from PC-3 and LNCaP human prostate carcinoma cell lines [52].

HSP70 is almost always a component of exosomal cargo regardless of the cellular source of the exosomes and studies have suggested that the exosomal pathway may be a major mechanism for HSP70 secretion [27, 28, 54]. To analyze whether eHSP70 was secreted by LPS-stimulated THP1 cells via the exosomal pathway, we analyzed HSP70 content in the exosome and exosome-depleted fractions from LPS/FRH-exposed THP1 cell conditioned media and found HSP70 to be predominantly in the exosome-free fractions. The relatively low levels of HSP70 in the exosome fraction appears to be the constitutive form as it was detected in immunoblots using an antibody that recognized both inducible HSP72 and constitutive HSC70 forms but not with an antibody specific for the inducible form. This analysis showed that LPS treatment in 37°C THP1 cells increased the exosomal HSC70 content without increasing the number of exosomes released as detected by CD63 immunoblotting. Incubation at 39.5°C with or without LPS stimulation increased exosome release, but exposure to 39.5°C was not sufficient to increase the HSC70 content in the absence of LPS. These results suggest that LPS activation and FRH exposure exert independent effects on release of exosomal HSC70. FRH increases exosomal release while LPS increases the exosomal content of HSC70 but not inducible HSP72.

Many studies have demonstrated that increasing intracellular levels of HSP70 is cytoprotective in many tissues, including heart, lung and the brain [13, 55]. There is also unambiguous evidence that extracellular HSP70 exerts potent proinflammatory actions by activating TLR4 receptors on macrophages, dendritic cells, and NK cells [56–59]. Patients with severe sepsis and septic shock exhibit both higher levels of intracellular HSP70 in peripheral blood mononuclear cells and elevated levels of extracellular HSP70 [60]. The results of the present study and earlier studies [8, 21] demonstrate that exposure to temperatures reached during fever stimulates HSP70 expression and co-exposure to TLR agonists increases both HSP70 synthesis and secretion. Based on the biological actions of intra- and extracellular HSP70, it may be beneficial to maximize intracellular HSP70 expression while reducing its secretion. The THP1 data from this study suggest that removing the FRH component by treating or suppressing fever would only modestly reduce HSP70 release and completely eliminate the increase in intracellular HSP70. On the other hand blocking TLR signaling would eliminate HSP70 secretion without affecting the increase in extracellular levels. However, it is unknown whether the findings in cell culture can be extrapolated to patients, thus underscoring the importance of future studies in animal models and humans trials.
